# Supplementary material for: HMGA2 directly mediates chromatin condensation in association with neuronal fate regulation
Source: Nat Commun. 2023 Oct 12;14:6420. doi: 10.1038/s41467-023-42094-9 (PMC10570362; doi:10.1038/s41467-023-42094-9)
Supplement: Supplementary file 1 — Supplementary Information [file 41467_2023_42094_MOESM1_ESM.pdf]

Supplementary figures

Supplementary figure 1

a IP-MS, Neuro2A cells

Protein	IgG		HMGA2 IP	
	Score	PSM	Score	PSM
Histone H2B type 1-F/J/L	232.62	7	1249.32	30
Histone H1.2	456.80	14	631.61	19
Histone H1.4	340.88	13	567.06	19
High-mobility group protein HMGI-C	32.13	1	499.06	21
Histone H1.1	138.96	6	488.87	16
Histone H1.5	317.02	10	449.66	14
Histone H2A type 1-H	91.97	4	325.86	10
Histone H2A type 2-C	91.97	4	270.33	9
Histone H1.0	60.34	1	181.52	6
40S ribosomal protein SA	38.11	1	149.76	6
Synaptotagmin-like protein 4	73.70	2	105.94	4
Histone H2AX			93.91	3
Mediator of DNA damage checkpoint protein 1			86.40	3
Histone H2A.Z			84.42	4

b

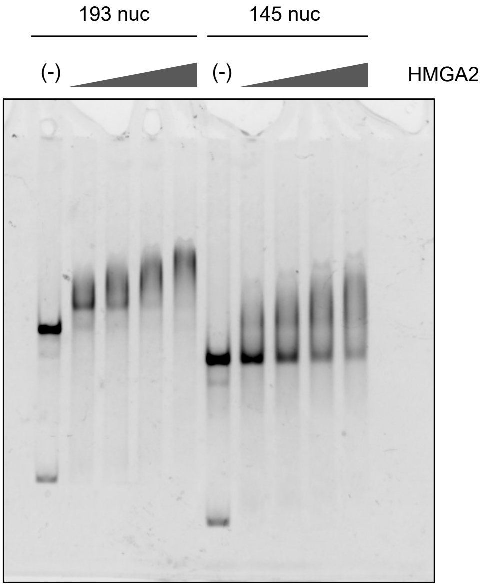

Supplementary Fig. 1. HMGA2 forms a complex with histone H1

(a) A chromatin fraction of Neuro2A cells overexpressing mouse HMGA2 was subjected to immunoprecipitation with antibodies to HMGA2 or control IgG, and the resulting precipitates were subjected to MS analysis. Data are from one experiment.

(b) EMSA analysis of incubation mixtures containing increasing amounts of HMGA2 (0, 0.15, 0.25, 0.35, 0.45 μM) together with mononucleosomes (0.1 μM) which have different length (193 or 145 nucleic acids) of linker DNA. The native PAGE gel was stained with ethidium bromide to detect DNA.

Supplementary figure 2

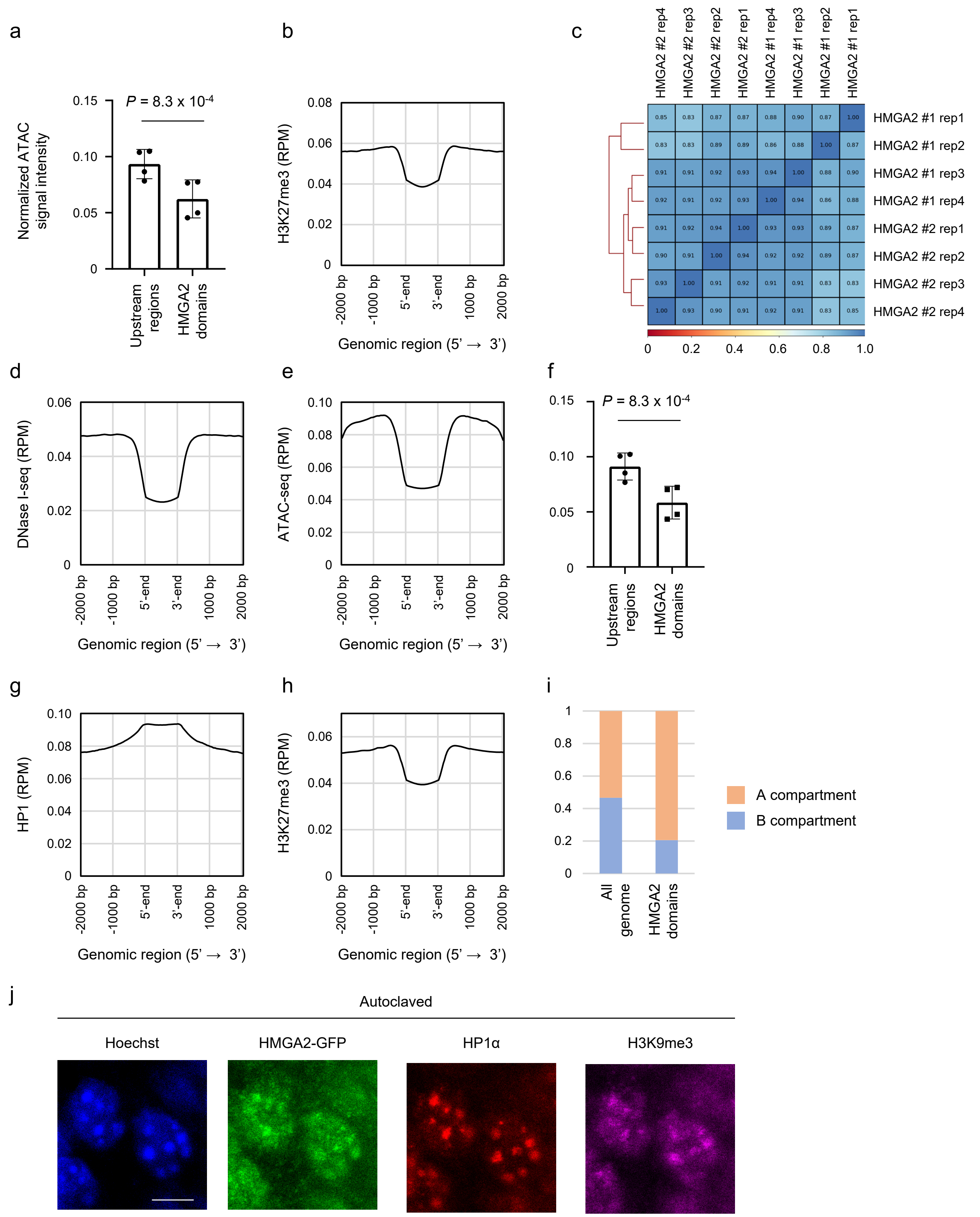

Supplementary Fig. 2. HMGA2 preferentially localizes to heterochromatin in vivo

(a) Quantification of ATAC-seq signals at HMGA2 domains and their upstream (–2 kbp to 0) regions shown in Fig. 3d. Data are means ± s.d. (n = 4 independent experiments). The P value was determined with the two-tailed Student's t test.

(b) Representative plot of H3K27me3 signals (RPM) around HMGA2 domains. HMGA2 domains (5' to 3') are indicated at the center of the x-axis.

(c) Correlation of HMGA2 ChIP-seq signals with different HMGA2 antibodies.

(d-i) DNase I–seq (d), ATAC-seq (e, f), HP1 (g), H3K27me3 (h), Hi-C (i) results are shown for experiments similar to those in Fig. 3b-e but for which ChIP-seq was performed with an in-house preparation of antibodies to HMGA2 instead of with commercial antibodies.

(j) Fluorescence microscopy of a section of the *Hmga2-EGFP* mouse neocortex at E11.5 showing Hoechst 33342, HP1α and H3K9me3 staining (scale bars = 5 μm).

Supplementary figure 3

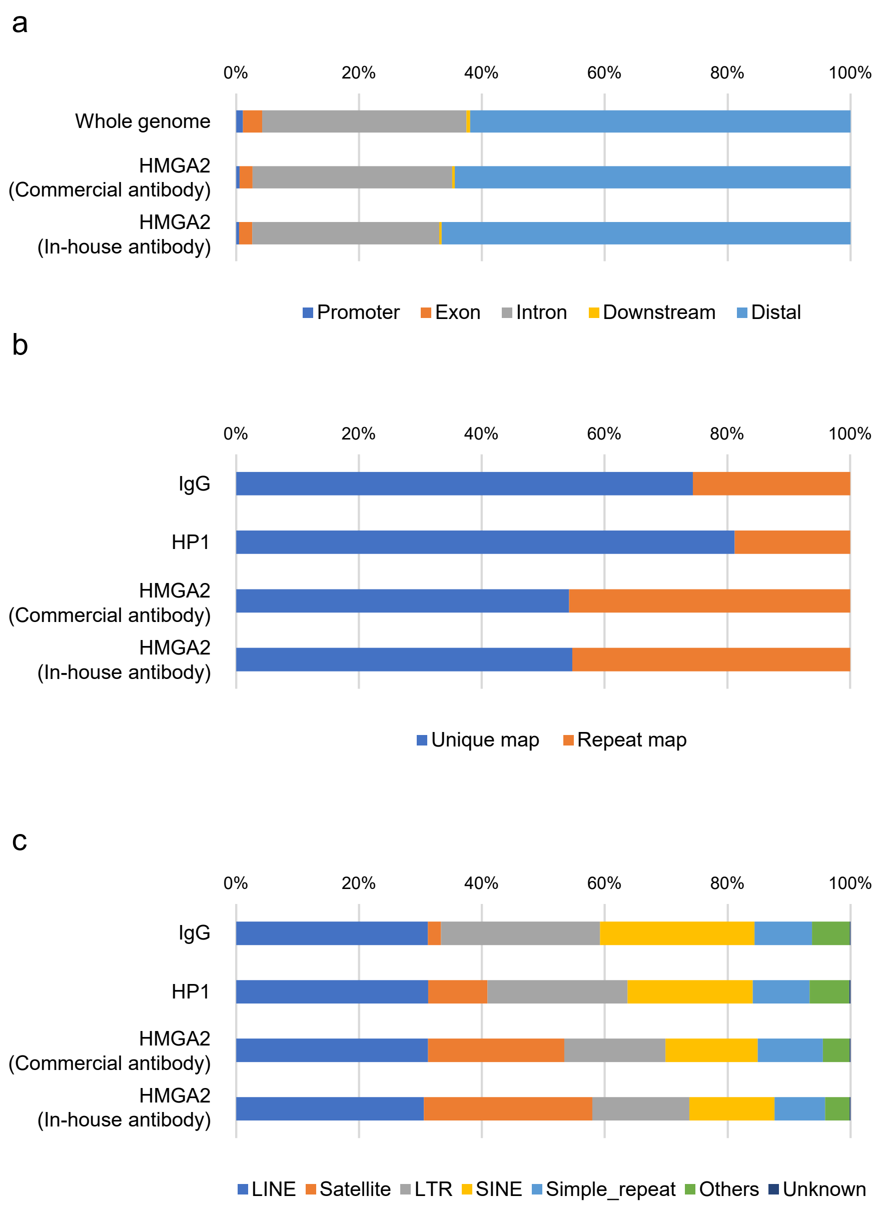

Supplementary Fig. 3. Genomic locations of HMGA2 peaks

(a) Percentages of HGMA2 domains localized to promoters (- 1000 bp to 0 bp from TSS), exons, introns, downstream (0 bp to + 1000 bp from TTS) and distal regions as a comparison of those of the mouse whole genome (mm10).

(b, c) Percentage of HGMA2 domains localized to repetitive elements.

Supplementary figure 4

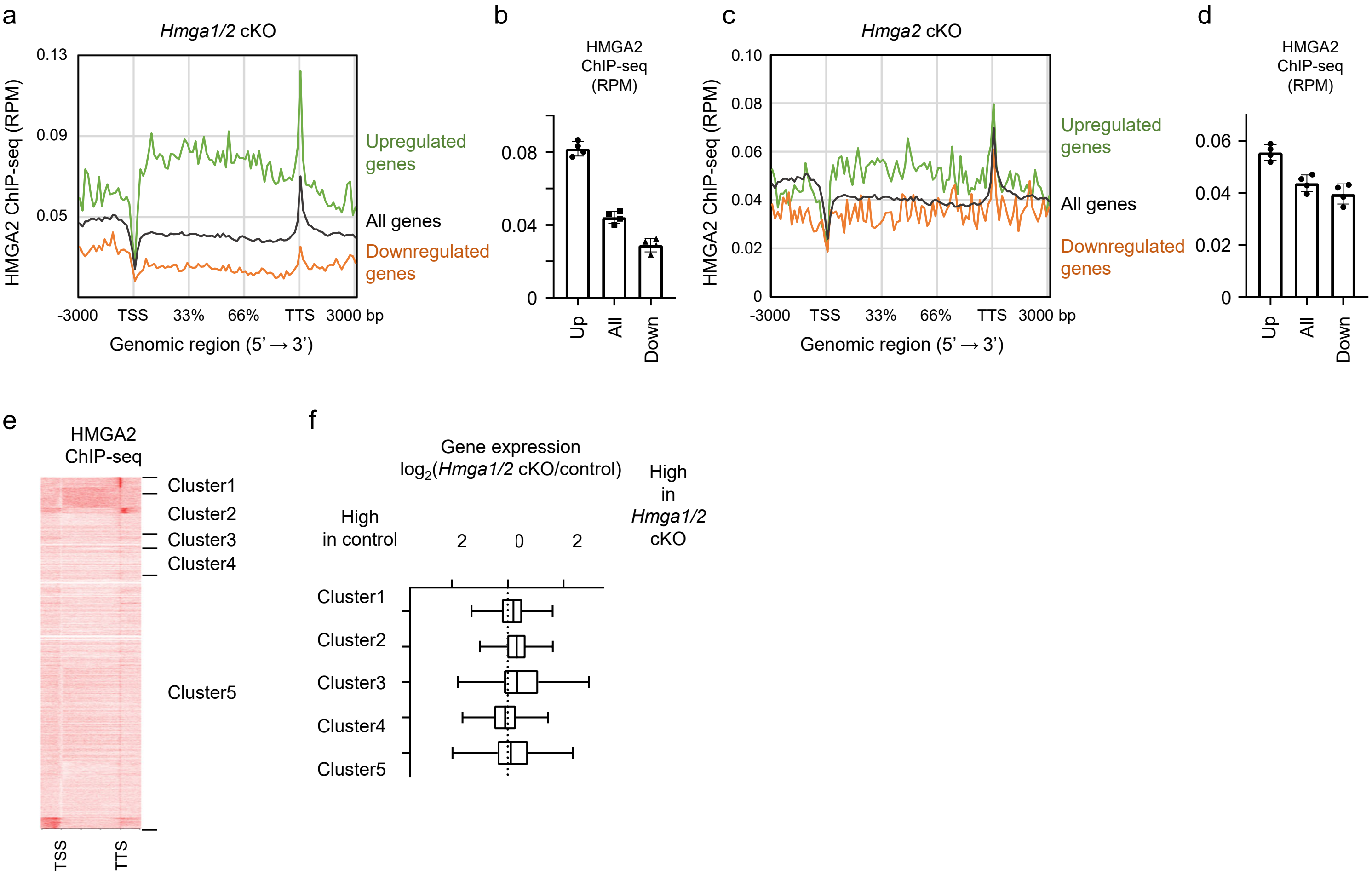

Supplementary Fig. 4. Reproducibility of data in Fig. 5.

The analyses presented in Fig. 4 were repeated with ChIP-seq results obtained with an in-house preparation of antibodies to HMGA2 instead of with commercial antibodies.

(a) Representative plot of averaged HMGA2 ChIP-seq signals around the gene body of all genes (black), genes upregulated by *Hmga1/2* cKO (green, 1266 genes), and genes downregulated by *Hmga1/2* cKO (orange, 1826 genes). TSS, transcription start site; TTS, transcription termination site.

(b) Average signals at the gene body were calculated for each gene set in (a). Data are means ± s.d. (n = 4 independent experiments). Tukey's multiple comparison test.

(c) Representative plot of averaged HMGA2 ChIP-seq signals around the gene body of all genes (black), genes upregulated by *Hmga2* cKO (green, 518 genes), and genes downregulated by *Hmga2* cKO (orange, 341 genes).

(d) Average signals at the gene body were calculated for each gene set in (c). Data are mean ± s.d. (n = 4 independent experiments). Tukey's multiple comparison test.

(e) HMGA2 binding patterns were clustered by ngsplot (k-means clustering, k = 5). The HMGA2 ChIP-seq signals ranging from 3000 bp upstream of the TSS to 3000 bp downstream of the TTS are shown as a heat map.

(f) Differences in gene expression for each cluster between neocortical NPCs of *Hmga1/2* cKO mice and those of control mice. Data are presented as box plots, with the boxes representing the median and upper and lower quartiles and the whiskers indicating the range. (n = 3 independent experiments)

Supplementary figure 5

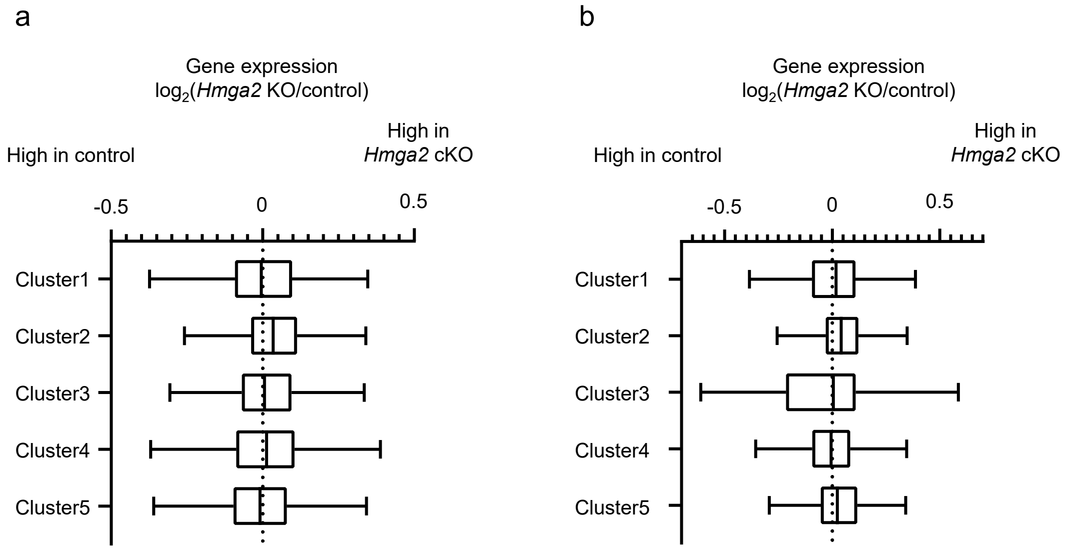

Supplementary Fig. 5. Gene body localization of HMGA2 is associated with gene repression.
(a,b) Differences in gene expression for each cluster between neocortical NPCs of *Hmga2* cKO mice and those of control mice. Data are presented as box plots, with the boxes representing the median and upper and lower quartiles and the whiskers indicating the range. HMGA2 ChIP-seq were conducted with commercial antibodies (a) or in-house antibodies (b).

Supplementary figure 6

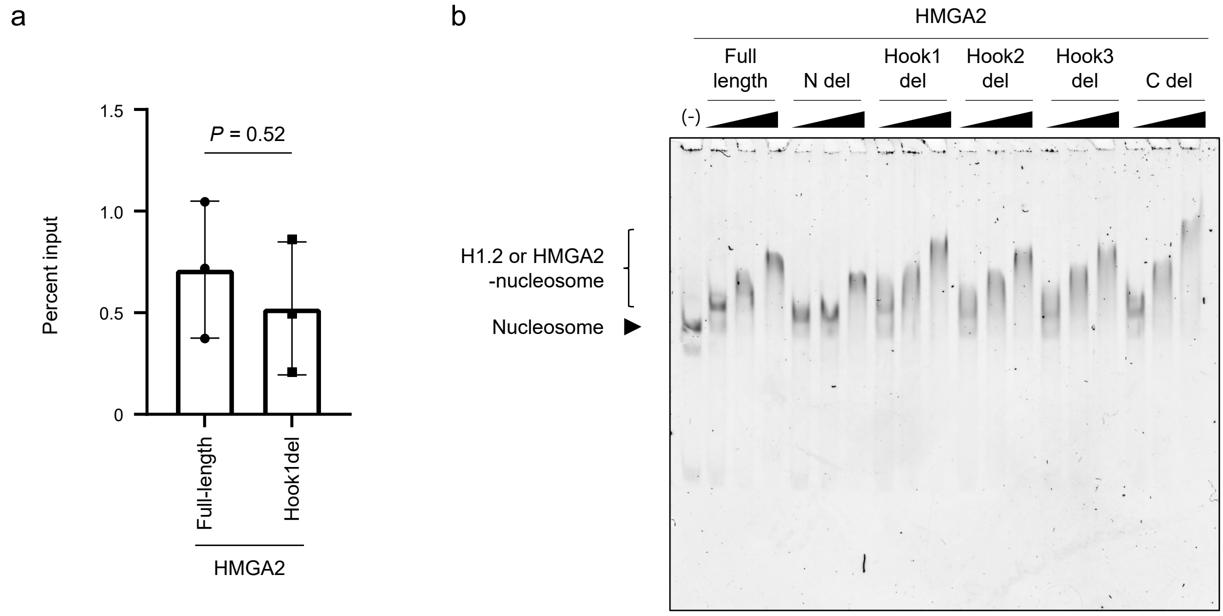

Supplementary Fig. 6. Full-length and hook1 del forms of HMGA2 bind to nucleosomes in vivo and in vitro.

(a) ChIP analysis of Full-length and hook1 del forms of HMGA2. E16.5 neocortical cells after in utero electroporation at E15.5 were subjected to ChIP analysis and the amount of immunoprecipitated DNA was measured. Data are means ± s.d. (n = 3 independent experiments). The P value was determined with the two-tailed Student's t test.

(b) Various amounts of recombinant human H1.2 or of mutant forms of HMGA2 were subjected to EMSA analysis as in Fig. 1c with mononucleosomes labeled with Cy3 and Cy5 as in Fig. 5d.

Supplementary figure 7

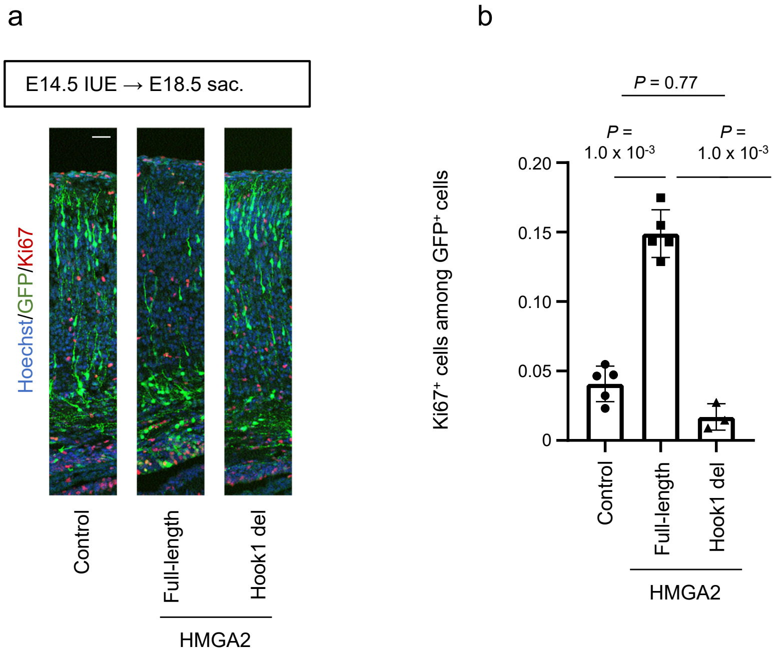

Supplementary Fig. 7. HMGA2 regulates neuronal cell fate in an AT-hook 1–dependent manner.
(a) Immunohistofluorescence analysis of GFP and Ki67 in the neocortex of mice at E18.5 after IUE at E14.5. Scale bars, 30 µm.
(b) Proportion of $Ki67^+$ cells among GFP^+ cells in images as in (a). Data are means ± s.d. (n = 3 to 5 independent experiments). The P values were determined by one-way ANOVA followed by Tukey's multiple comparison test.

Supplementary figure 8

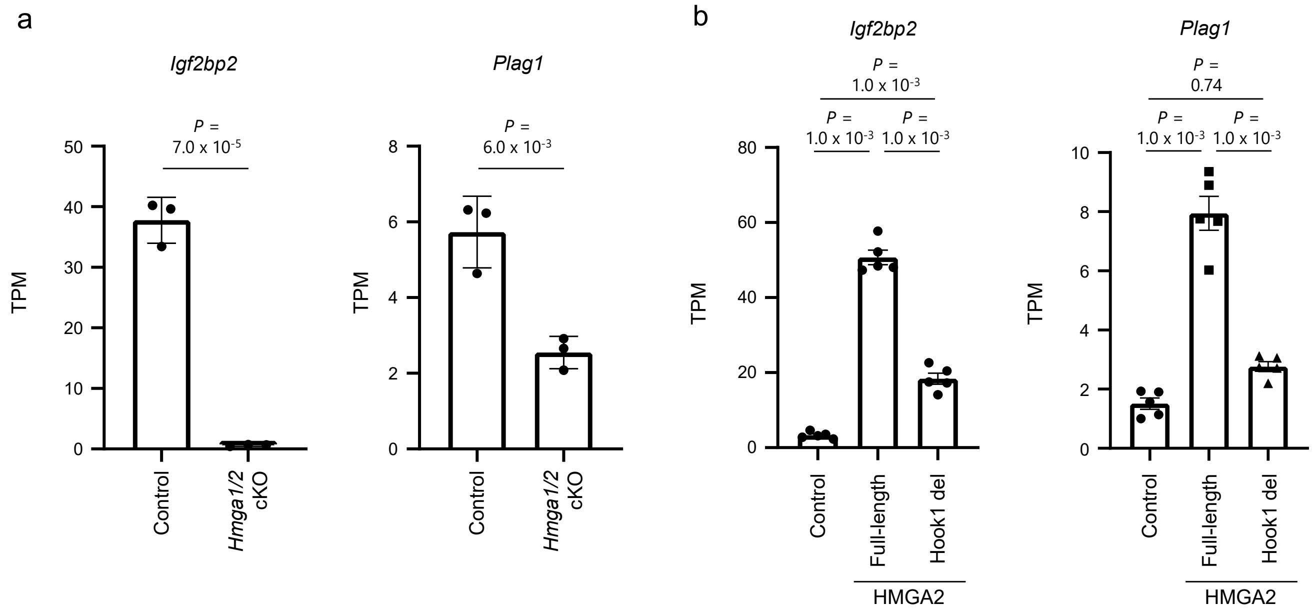

Supplementary Fig. 8. Effects of HMGA2 on expression of *Igf2bp2* and *Plag1*.

(a) Expression levels of *Igf2bp2* and *Plag1* in RNA-seq analysis of $CD133^{high}$ cells isolated from the neocortex of *Hmga1/2* cKO mice at E12.5. Data are means ± s.d. ($n = 3$ independent experiments). The *P* values were determined with the two-tailed Student's *t* test.

(b) Expression levels of *Igf2bp2* and *Plag1* in RNA-seq analysis of $CD133^{high}CD24^{low}$ cells isolated from the mouse neocortex at P1 after IUE at E15.5 as in Fig. 7f. Data are means ± s.d. ($n = 5$ independent experiments). The *P* values were determined by one-way ANOVA followed by Tukey's multiple comparison test.

Supplementary figure 9

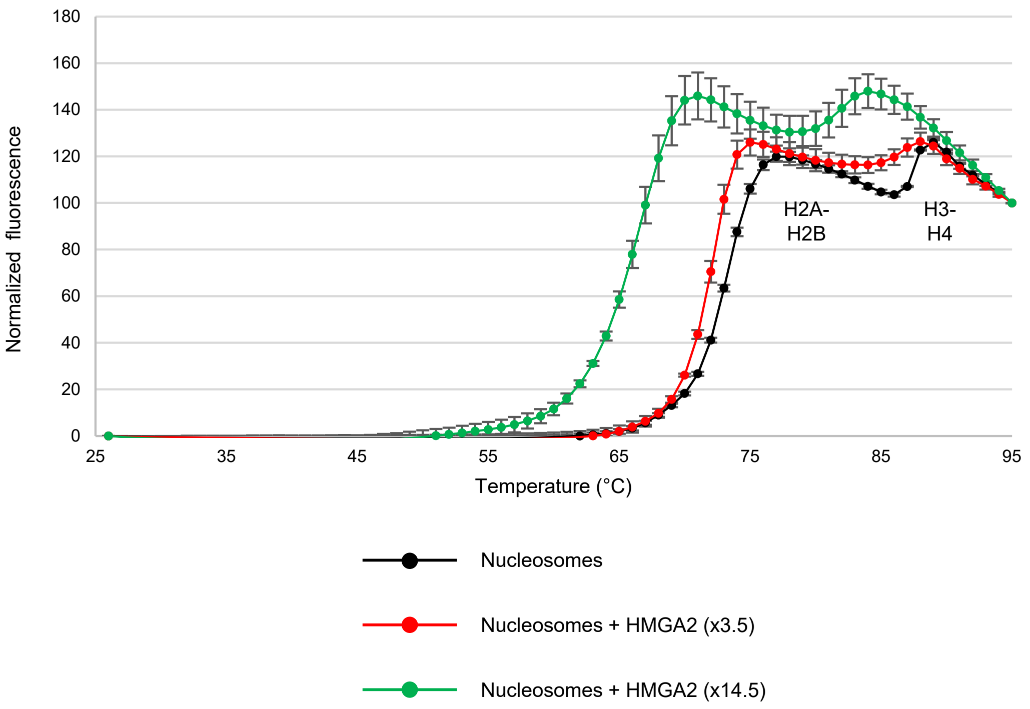

Supplementary Fig. 9. Effect of HMGA2 on thermal stability of nucleosomes.
Normalized fluorescence intensity curves are shown for the thermal disruption of nucleosomes in the absence or presence of HMGA2. The first and second phases correspond to the H2A-H2B dissociation and the H3-H4 dissociation from the nucleosome, respectively. Data are means ± s.d. (n = 3 independent experiments).

Supplementary figure 10

Protein purification

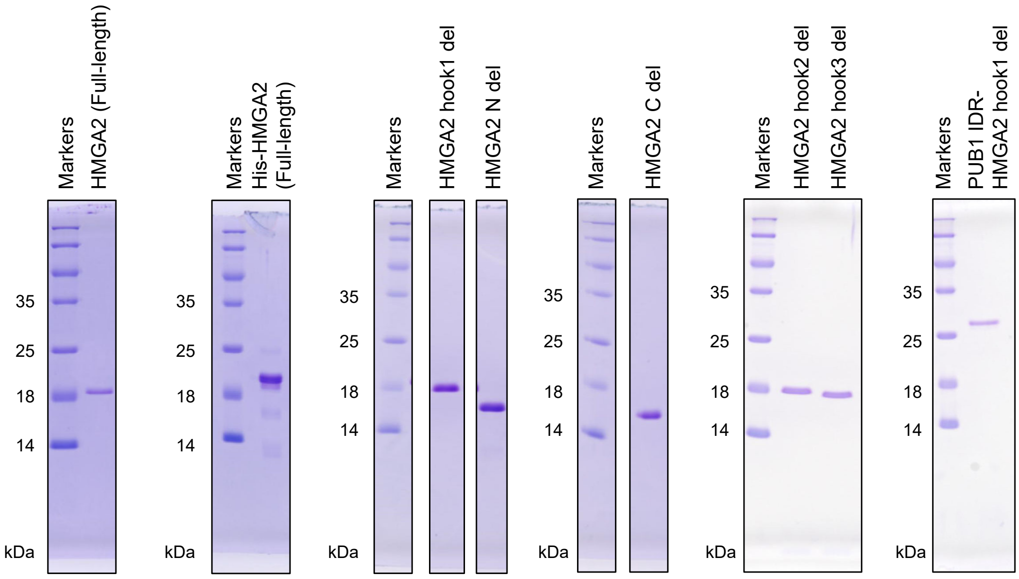

Fig.1b

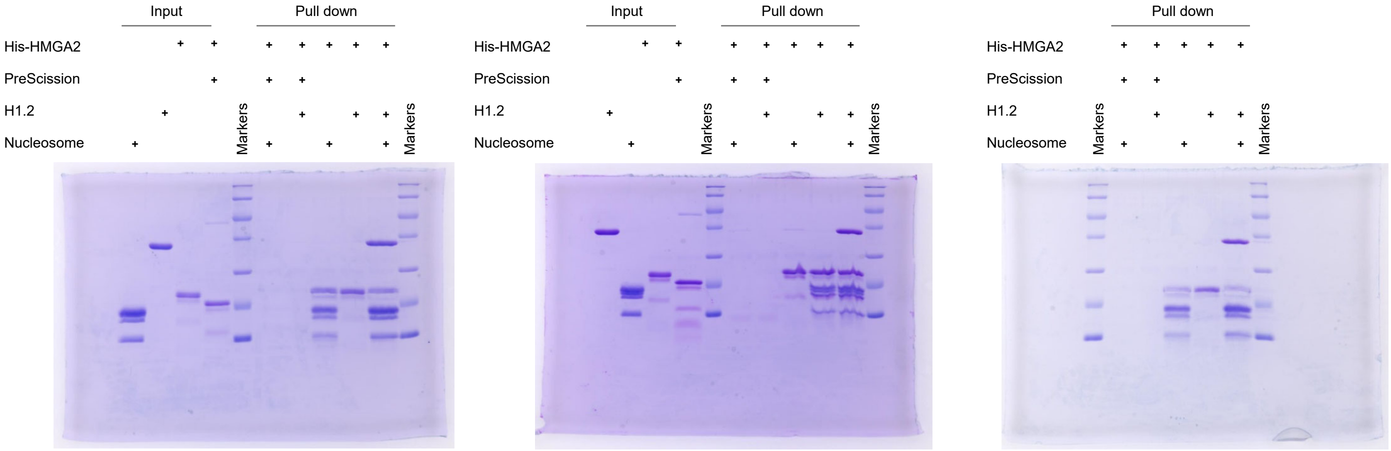

Fig.2b

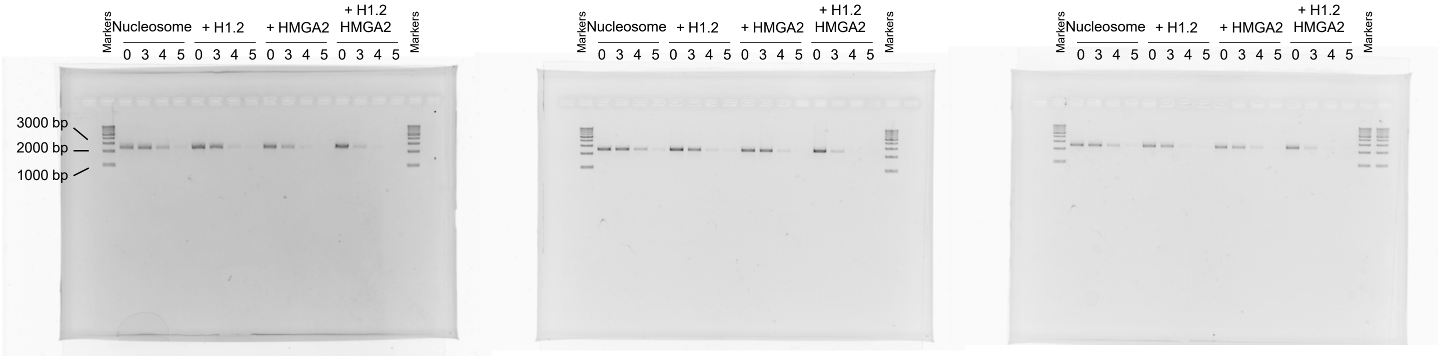

Fig.2e left

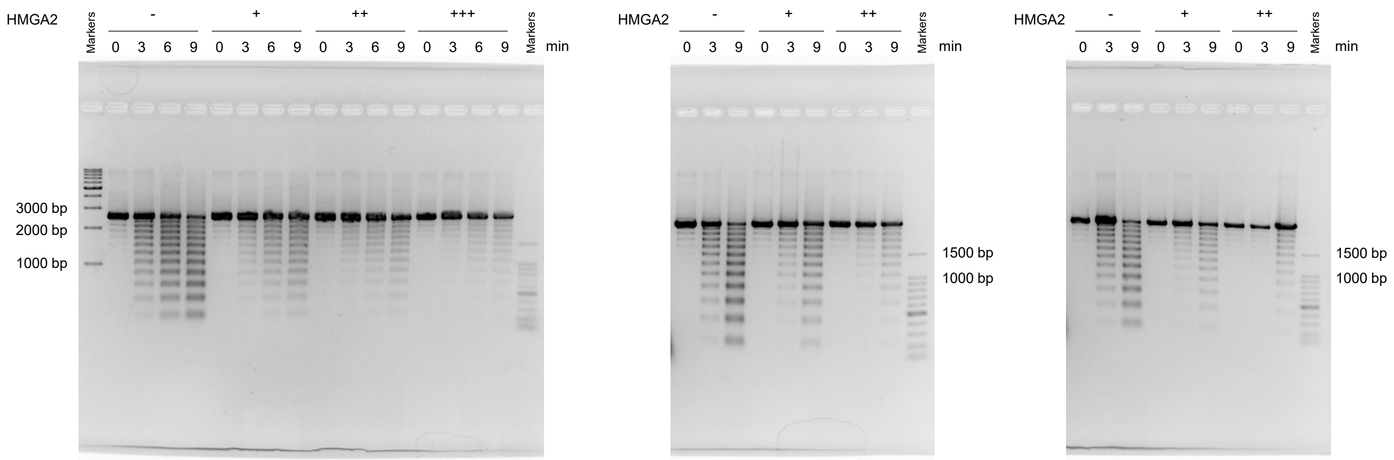

Fig.2e right

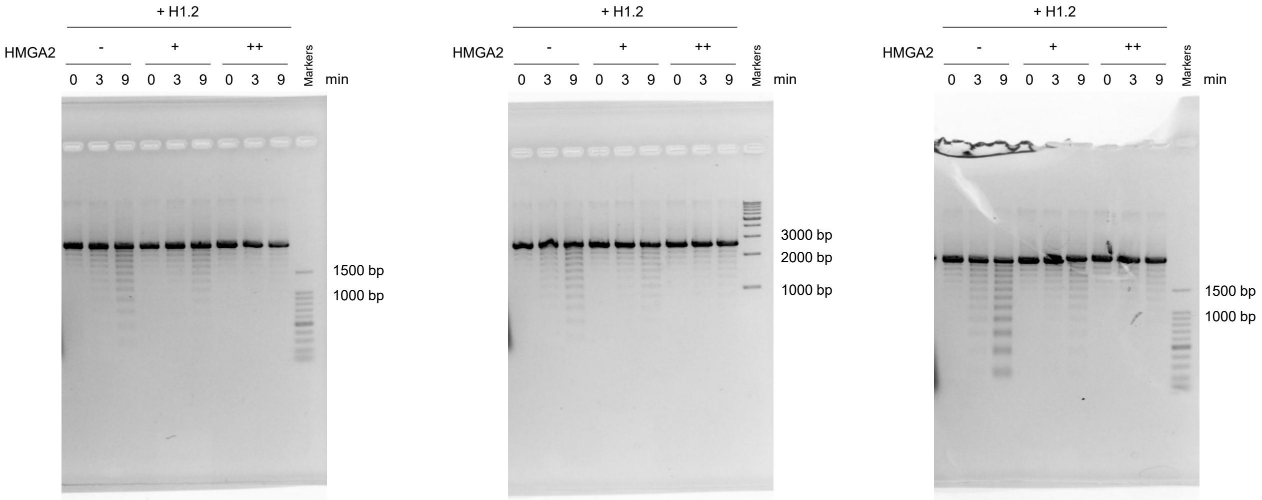

Fig.2f

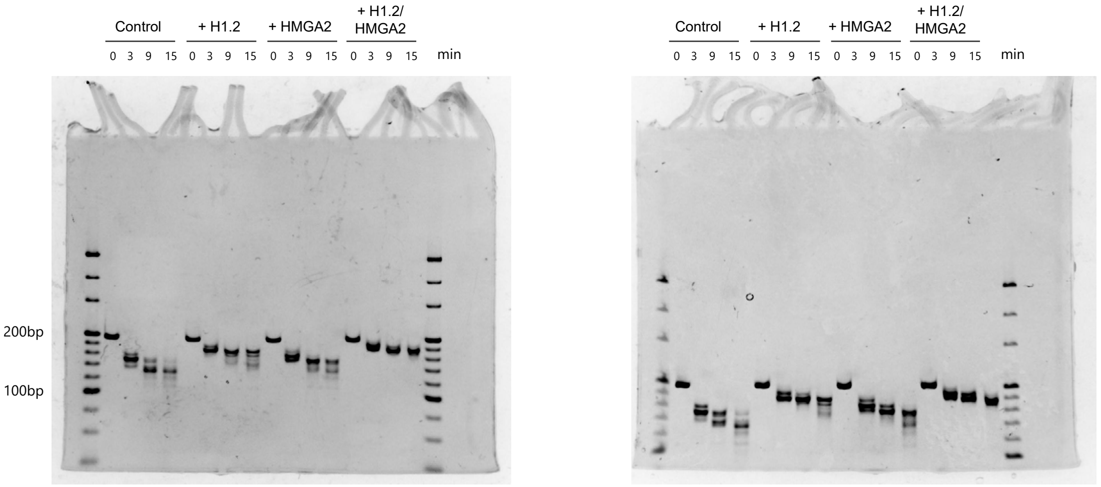

Fig.2e left

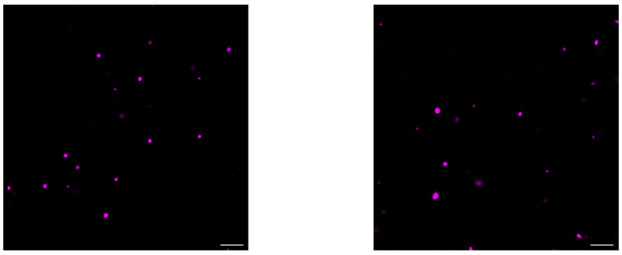

Fig.6b

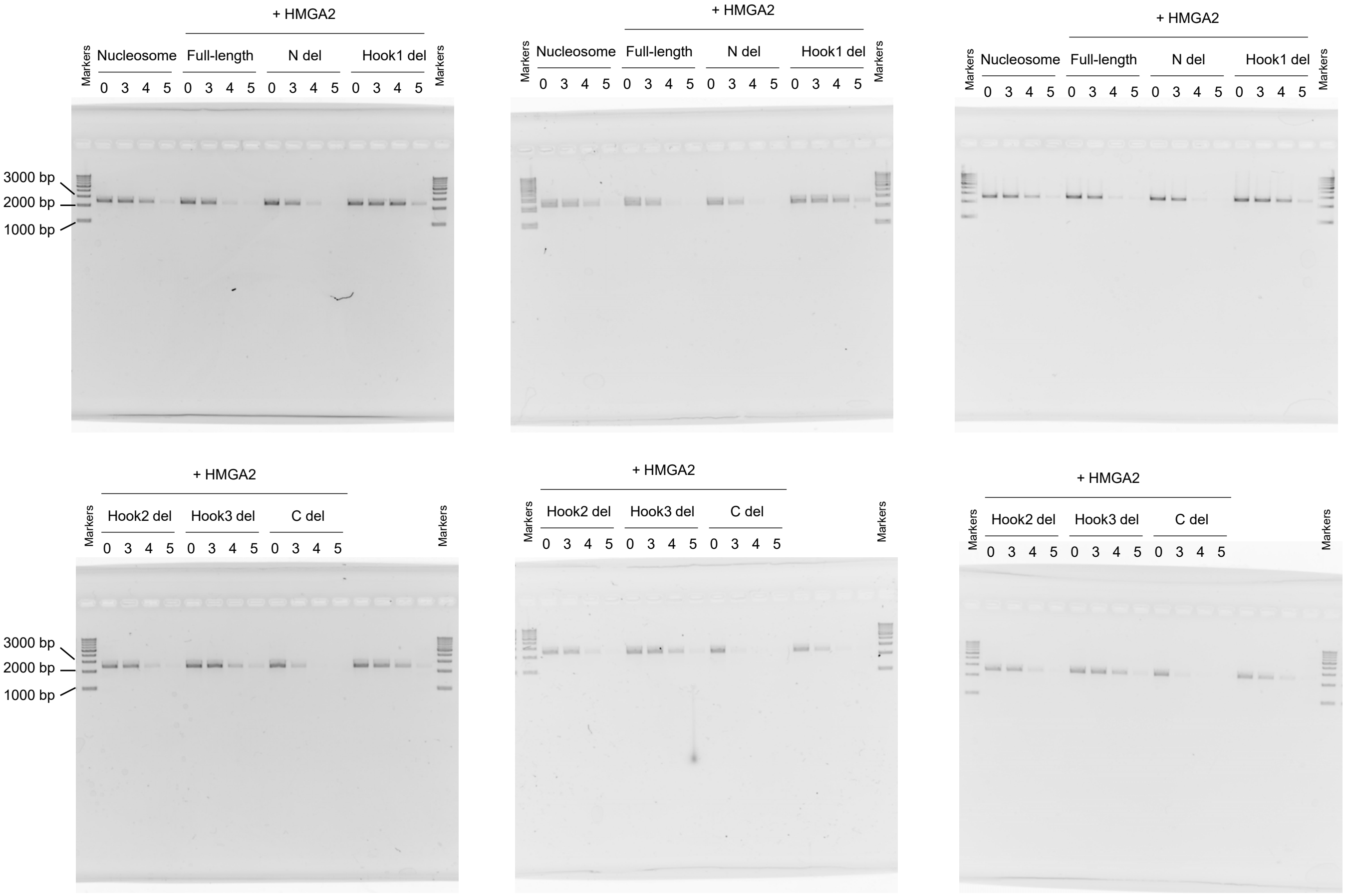

Fig.6c

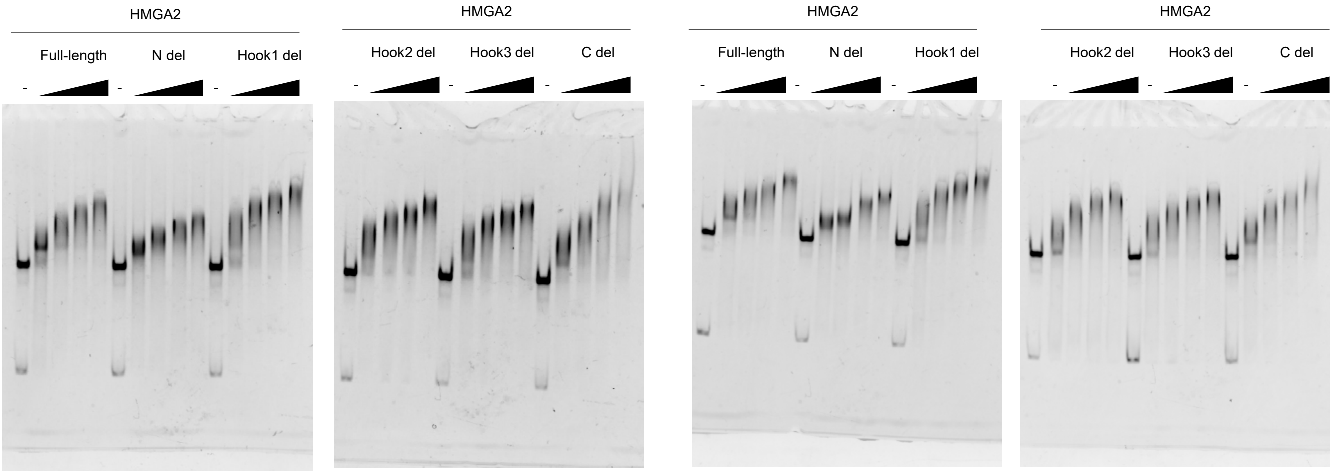

Supplementary Fig.6

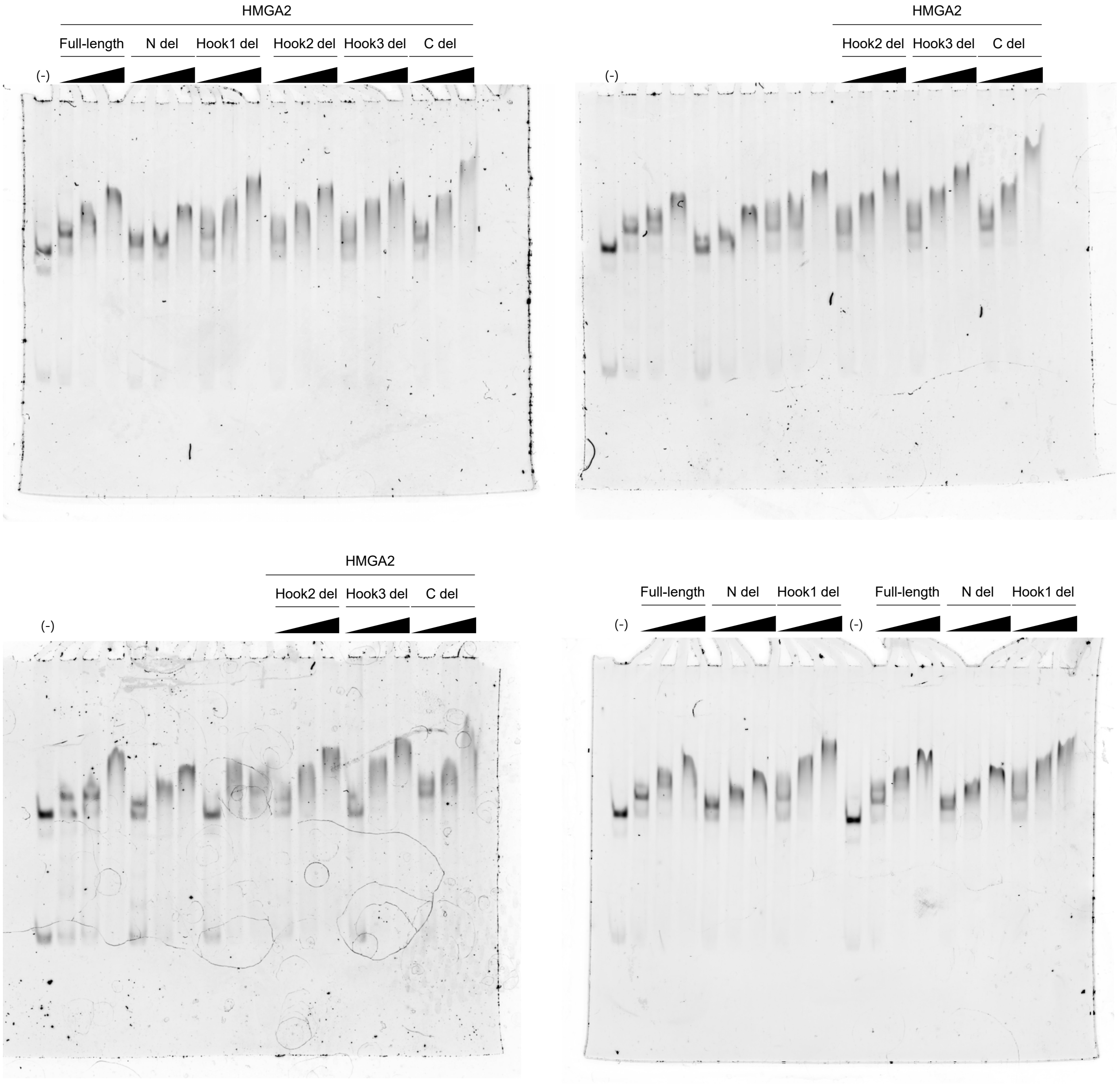

Supplementary Fig. 10. Protein purification, replicated experiments and full images of gels.
