# Supplementary material for: HMGA2 directly mediates chromatin condensation in association with neuronal fate regulation
Source: Nat Commun. 2023 Oct 12;14:6420. doi: 10.1038/s41467-023-42094-9 (PMC10570362; doi:10.1038/s41467-023-42094-9)
Supplement: Supplementary file 3 — Reporting Summary [file 41467_2023_42094_MOESM3_ESM.pdf]

nature portfolio

Corresponding author(s): Yukiko Gotoh

Last updated by author(s): Aug 22, 2023

Reporting Summary

Nature Portfolio wishes to improve the reproducibility of the work that we publish. This form provides structure for consistency and transparency in reporting. For further information on Nature Portfolio policies, see our Editorial Policies and the Editorial Policy Checklist.

Statistics

For all statistical analyses, confirm that the following items are present in the figure legend, table legend, main text, or Methods section.

n/a	Confirmed	
☐	☒	The exact sample size (*n*) for each experimental group/condition, given as a discrete number and unit of measurement
☐	☒	A statement on whether measurements were taken from distinct samples or whether the same sample was measured repeatedly
☐	☒	The statistical test(s) used AND whether they are one- or two-sided<br>*Only common tests should be described solely by name; describe more complex techniques in the Methods section.*
☒	☐	A description of all covariates tested
☐	☒	A description of any assumptions or corrections, such as tests of normality and adjustment for multiple comparisons
☐	☒	A full description of the statistical parameters including central tendency (e.g. means) or other basic estimates (e.g. regression coefficient) AND variation (e.g. standard deviation) or associated estimates of uncertainty (e.g. confidence intervals)
☐	☒	For null hypothesis testing, the test statistic (e.g. *F*, *t*, *r*) with confidence intervals, effect sizes, degrees of freedom and *P* value noted<br>*Give P values as exact values whenever suitable.*
☒	☐	For Bayesian analysis, information on the choice of priors and Markov chain Monte Carlo settings
☒	☐	For hierarchical and complex designs, identification of the appropriate level for tests and full reporting of outcomes
☒	☐	Estimates of effect sizes (e.g. Cohen's *d*, Pearson's *r*), indicating how they were calculated

Our web collection on statistics for biologists contains articles on many of the points above.

Software and code

Policy information about availability of computer code

Data collection	Imaging: Leica SPS confocal microscope, Axiovert 200M microdscope (Zwiss), LAS4000 image analyzer (GE Healthcare) or Amersham Imager 680 QC (GE Healthcare) Sequenecing analysis: Hiseq2000/2500/3000/X (Illumina) AFM: NanoWizard IIR instrument (JPK)MS: Advance Ultra High Performance Liquid Chromatography system (AMR/Michrom Bioscience), Q Exactive mass spectrometer (Thermo Fisher) Turbidity: Nano-drop ND-1000 instrument (Thermo Fisher)
Data analysis	Data analysis methods were described at Methods section. No custom codes have been developed in this study.<br>MS: Proteome Discoverer version 1.4 (Thermo Fisher), Mascot search engine version 2.5 (Matrix Science) Imaging: ImageJ (U.S. National Institutes of Health [NIH]) RNA-seq: trimmomatic (0.39), Hisat2(2.2.1), featureCounts (2.0.1), RUVSeq (1.34.0) ATAC-seq, DNase-seq, ChIP-seq: bowtie2 (2.4.5), samtools (1.14), F-seq, ngsplot (2.63), deeptools (3.5.1), RepEnrich2 Hi-C: Hi-C juicer, HiCExplorer(3.7)

For manuscripts utilizing custom algorithms or software that are central to the research but not yet described in published literature, software must be made available to editors and reviewers. We strongly encourage code deposition in a community repository (e.g. GitHub). See the Nature Portfolio guidelines for submitting code & software for further information.

Data

Policy information about availability of data

All manuscripts must include a data availability statement. This statement should provide the following information, where applicable:

- Accession codes, unique identifiers, or web links for publicly available datasets
- A description of any restrictions on data availability
- For clinical datasets or third party data, please ensure that the statement adheres to our policy

The data and codes that support this study are available from the corresponding authors upon reasonable request. The sequence data have been deposited in the DNA Data Bank of Japan (DDBJ) Sequence Read Archive under the following accession codes: DRA008363, DRA008364, DRA010294, DRA010295 (HMGA2 ChIP-seq), DRA015284 (DNase I-seq), DRA015285 (ATAC-seq), DRA015234 (Hi-C), DRA008751 (H3K27me3 ChIP-seq) and DRA015260, DRA015261, DRA015262, DRA016538 (RNA-seq). Supplementary files have also been deposited in the DDBJ Genomics Expression Archives under the accession code E-GEAD-571, E-GEAD-572, E-GEAD-573, E-GEAD-574, E-GEAD-575, E-GEAD-576, E-GEAD-577, E-GEAD-578, E-GEAD-581, E-GEAD-582, E-GEAD-624 (https://ddbj.nig.ac.jp/public/ddbj_database/gea/experiment/E-GEAD-000/). Source data are provided with this paper.
Sequences were mapped to the reference mouse genome (mm10).

Research involving human participants, their data, or biological material

Policy information about studies with human participants or human data. See also policy information about sex, gender (identity/presentation), and sexual orientation and race, ethnicity and racism.

Reporting on sex and gender	N/A
Reporting on race, ethnicity, or other socially relevant groupings	N/A
Population characteristics	N/A
Recruitment	N/A
Ethics oversight	N/A

Note that full information on the approval of the study protocol must also be provided in the manuscript.

Field-specific reporting

Please select the one below that is the best fit for your research. If you are not sure, read the appropriate sections before making your selection.

[x] Life sciences [ ] Behavioural & social sciences [ ] Ecological, evolutionary & environmental sciences

For a reference copy of the document with all sections, see nature.com/documents/nr-reporting-summary-flat.pdf

Life sciences study design

All studies must disclose on these points even when the disclosure is negative.

Sample size	Sample size was predetermined based on literature data using the same well established experimental approaches (Hirabayashi et al., Nauron, 2009; Eto et al., Nature Communications, 2020).
Data exclusions	No data exclustion
Replication	We confirmed that all experiments in this study were replicated successfully. Figure legends, Supplementary figure 10 and Source data contain exact number of samples and animals used in this study.
Randomization	All sample allocation were randomized.
Blinding	All data were analyzed under blinded conditions. All data were collected under unblinded condition because of practical reason.

Reporting for specific materials, systems and methods

We require information from authors about some types of materials, experimental systems and methods used in many studies. Here, indicate whether each material, system or method listed is relevant to your study. If you are not sure if a list item applies to your research, read the appropriate section before selecting a response.

Materials & experimental systems

n/a	Involved in the study
☐	☒ Antibodies
☐	☒ Eukaryotic cell lines
☒	☐ Palaeontology and archaeology
☐	☒ Animals and other organisms
☒	☐ Clinical data
☒	☐ Dual use research of concern
☒	☐ Plants

Methods

n/a	Involved in the study
☐	☒ ChIP-seq
☒	☐ Flow cytometry
☒	☐ MRI-based neuroimaging

Antibodies

Antibodies used	Chicken anti-GFP (1:1000 dilution, Abeam ab13970)<br>Rat anti-GFP (1:1000 dilution, Nacalai Tesque GF090R)<br>Rabbit anti-GFP (1:1000 dilution, MBL 598)<br>Rabbit anti-Sox2 (1:200 dilution, Cell Signaling 3728)<br>Chicken anti-Tbr2 (1:500 dilution, Millipore AB15894)<br>Goat anti-NeuroDl (1:100 dilution, Santa Cruz Biotechnology sc-1084)<br>Rabbit anti-Ki67 (1:500 dilution, Abcam ab16667)<br>Rabbit HP1a (1:500 dilution, CST 26165)<br>Mouse H3K9me3 (1:800 dilution, Thermo Fisher MABI 0319)<br>Rabbit HMGA2 (1:500 dilution, Cell signaling 8179 or in-house)<br><br>Donkey anti-Rat IgG (H+L) Highly Cross-Adsorbed Secondary Antibody, Alexa Fluor 488 (A-21208) (1:1000 dilution)<br>Donkey anti-Rabbit IgG (H+L) Highly Cross-Adsorbed Secondary Antibody, Alexa Fluor 488 (A-21206) (1:1000 dilution)<br>Donkey anti-Rabbit IgG (H+L) Highly Cross-Adsorbed Secondary Antibody, Alexa Fluor 555 (A-31572) (1:1000 dilution)<br>Donkey anti-Mouse IgG (H+L) Highly Cross-Adsorbed Secondary Antibody, Alexa Fluor 555 (A-31570) (1:1000 dilution)<br>Donkey anti-Goat IgG (H+L) Highly Cross-Adsorbed Secondary Antibody, Alexa Fluor 555 (A-21432) (1:1000 dilution)<br>Donkey anti-Rabbit IgG (H+L) Highly Cross-Adsorbed Secondary Antibody, Alexa Fluor 647 (A-31573) (1:1000 dilution)<br>AlexaFluor 488 Affini Pure Donkey Anti-Chicken IgY (IgG) (H+L) antibody Jackson Immuno Research (703-545-155) (1:1000 dilution)
Validation	Yuizumi et al., Stem Cells, 2021; Fang et al., Frontiers in Neuroscience, 2023; Harada et al., Nature Communications, 2021; Fujii et al., Genes to Cells, 2013<br>https://www.cellsignal.com/products/primary-antibodies/hpla-antibody/2616<br>https://www.thermofisher.com/antibody/product/Histone-H3K9me3-Antibody-clone-MAB 1-0319-Monoclona1/61013

Eukaryotic cell lines

Policy information about cell lines and Sex and Gender in Research

Cell line source(s)	Neuro2A (provided by Dr. Hidenori Ichijo, the University of Tokyo)<br>IMR90 (provided by Dr. Nobuyoshi Akimitsu, the University of Tokyo)
Authentication	The authors declare that the cell lines were authenticated based on their morphology
Mycoplasma contamination	The cell lines were tested negative for mycoplasma contamination.
Commonly misidentified lines (See ICLAC register)	N/A

Animals and other research organisms

Policy information about studies involving animals; ARRIVE guidelines recommended for reporting animal research, and Sex and Gender in Research

Laboratory animals	All animals are described in Methods section.
Wild animals	No study involvinf wild animals
Reporting on sex	Both male and female embryos were used.
Field-collected samples	No field collected samples were used in the study
Ethics oversight	All animals were maintained and studied according to protocols approved by the Animal Care and Use Committee of The University of Tokyo.

Note that full information on the approval of the study protocol must also be provided in the manuscript.

Plants

Seed stocks	N/A
Novel plant genotypes	N/A
Authentication	N/A

ChIP-seq

Data deposition

☒ Confirm that both raw and final processed data have been deposited in a public database such as GEO.

☒ Confirm that you have deposited or provided access to graph files (e.g. BED files) for the called peaks.

Data access links *May remain private before publication.*	DRA008363, DRA010294,DRA008364,DRA010295
Files in database submission	HMGA2 ChIP-seq
Genome browser session (e.g. UCSC)	BigWig files were deposited.<br>E-GEAD-575, E-GEAD-576, E-GEAD-577, E-GEAD-578

Methodology

Replicates	n=4, see supplementary figure2c
Sequencing depth	36 bp single end, more than 40 million reads on average
Antibodies	Cell signaling 8179 or in-house
Peak calling parameters	F-seq, t=7
Data quality	See figure 3b and supplementary figure 2, 3.
Software	ChIP-seq: bowtie2 (2.4.5), samtools (1.14), F-seq, ngsplot (2.63), deeptools (3.5.1), RepEnrich2
